# Mid-Term Outcomes After Conversion Procedures Following Laparoscopic Sleeve Gastrectomy

**DOI:** 10.1007/s11695-023-06734-9

**Published:** 2023-07-29

**Authors:** Amanda S. Dirnberger, Julian Süsstrunk, Romano Schneider, Adisa Poljo, Jennifer M. Klasen, Marc Slawik, Adrian T. Billeter, Beat P. Müller-Stich, Ralph Peterli, Marko Kraljević

**Affiliations:** 1 University Digestive Health Care Center Basel – Clarunis, CH-4002 Basel, Switzerland; 2 Interdisciplinary Center of Nutritional and Metabolic Diseases, St. Clara Hospital, CH-4058 Basel, Switzerland; 3 Department of Clinical Research, University of Basel, CH-4031 Basel, Switzerland

**Keywords:** Sleeve gastrectomy, Conversion, Insufficient weight loss, GERD

## Abstract

**Purpose:**

In the long term, laparoscopic sleeve gastrectomy (SG) may be associated with insufficient weight loss (IWL), gastroesophageal reflux disease (GERD), and persistence or relapse of associated medical problems. This study’s objective is to present mid-term results regarding weight loss (WL), evolution of associated medical problems, and reoperation rate of patients who underwent a conversion after SG.

**Methods:**

Retrospective single-center analysis of patients with a minimal follow-up of 2 years after conversion.

**Results:**

In this series of 549 SGs, 84 patients (15.3%) underwent a conversion, and 71 met inclusion criteria. They were converted to short biliopancreatic limb Roux-en-Y gastric bypass (short BPL RYGB) (*n* = 28, 39.4%), biliopancreatic diversion with duodenal switch (BPD/DS) (*n* = 19, 26.8%), long biliopancreatic limb Roux-en-Y gastric bypass (long BPL RYGB) (*n* = 17, 23.9%), and re-sleeve gastrectomy (RSG) (*n* = 7, 9.9%). Indications were GERD (*n* = 24, 33.8%), IWL (*n* = 23, 32.4%), IWL + GERD (*n* = 22, 31.0%), or stenosis/kinking of the sleeve (*n* = 2, 2.8%). The mean pre-revisional body mass index (BMI) was 38.0 ± 7.5 kg/m^2^. The mean follow-up time after conversion was 5.1 ± 3.1 years. The overall percentage of total weight loss (%TWL) was greatest after BPD/DS (36.6%) and long BPL RYGB (32.9%) compared to RSG (20.0%; *p* = 0.004; *p* = 0.049). In case of GERD, conversion to Roux-en-Y gastric bypass (RYGB) led to a resolution of symptoms in 79.5%. 16.9% of patients underwent an additional revisional procedure.

**Conclusion:**

In the event of IWL after SG, conversion to BPD/DS provides a significant and sustainable additional WL. Conversion to RYGB leads to a reliable symptom control in patients suffering from GERD after SG.

**Graphical Abstract:**

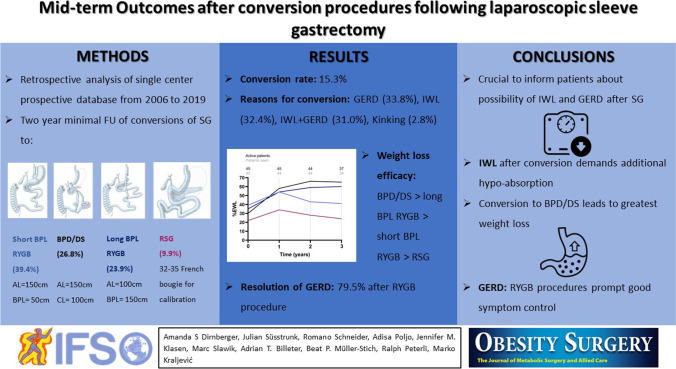

## Introduction

According to the 2018 International Federation for the Surgery of Obesity and Metabolic Disorders (IFSO) Survey, sleeve gastrectomy (SG) has become the most commonly performed bariatric procedure worldwide with 55.4% [1]. Primary SG is a widely accepted stand-alone procedure regarding weight loss (WL) with a good safety profile and a high rate of resolution of associated medical problems [2, 3]. However, different studies examining mid- to long-term results of SG have found potential associated complications. The most frequent are insufficient weight loss (IWL) or weight regain (WR), with studies showing WR rates of 27.8 to 51.4% after 7 years of follow-up, and gastroesophageal reflux disease (GERD), with an incidence of de novo GERD symptoms ranging from 23.0 to 42.9% within a 10-year follow-up period and an increase of postoperative GERD symptoms in 19.0% of patients [4–7]. Commonly found non-GERD-related persistent associated medical problems are arterial hypertension (HTN) and obstructive sleep apnea (20.2%) followed by type II diabetes (T2D) (12.8%) [8]. Multiple reasons have been attributed to IWL: technical factors such as enlarged postoperative sleeve volume due to insufficient resection as well as changes in dietary habits and physical activity [9, 10]. Further, postoperative sleeve dilatation may occur and is thought to promote WR, thus leading to the development of the banded SG [11, 12]. In regard to GERD, various underlying mechanisms have been described: lack of gastric compliance and emptying, a high-pressure setting after SG, an increased esophagogastric insertion angle, and a small gastric capacity [13–15]. Amongst others, these complications may necessitate a second operation. Conversion rates after SG range from 6.1 to 22.6% increasing with follow-up duration [16, 17]. The predominant causes found, were GERD (2.9 to 65.2%) and IWL/WR (11.8 to 55.7%) followed by stricture (14.0%), kinking of the sleeve (11.0%), or fistula (1.9%) [2, 4, 16–19]. The average time to conversion varied from 31 months after short- to 5.6 years after long-time follow-up [8, 18].

The objective of this study was to evaluate indications for conversion, weight loss outcomes, and postoperative complications as well as the evolution of associated medical problems and required further operations for patients who underwent a conversion after SG at a single institution.

## Methods

### Design, Patients, and Preoperative Workup

For this single-center retrospective study, data was obtained from a prospective database on patients undergoing bariatric surgery. Included were patients with a minimal follow-up of 24 months after conversion from SG. The study was approved by the local ethics committee. Reasons for conversion were: GERD in spite of medication and IWL with no fixed weight loss limit applied, including WR with relapse of associated medical problems and a combination of GERD + IWL as well as stenosis/kinking of the sleeve. In patients with IWL, we predominantly performed a biliopancreatic diversion with duodenal switch (BPD/DS) or long biliopancreatic limb Roux-en-Y gastric bypass (long BPL RYGB) if they additionally suffered from GERD, whereas if GERD was the main problem, a short biliopancreatic limb Roux-en-Y gastric bypass (short BPL RYGB) was chosen. Re-sleeve gastrectomy (RSG) was only considered when sleeve dilatation was confirmed. Prior to conversion, all patients were discussed by an interdisciplinary team of endocrinologists, nutritionists, psychiatrists, and bariatric surgeons and had a routine medical check-up. Additionally, an upper gastrointestinal series and endoscopy were performed in all patients to detect hiatal hernias and reflux esophagitis, in which case a hiatal hernia repair was performed during the conversion. Furthermore, an abdominal sonography was carried out in patients with intact gallbladder, and a concomitant cholecystectomy was performed in case of gallstones to prevent the risk of common bile duct stones (CBDS) and cholecystitis after a RYGB procedure and routinely during BPD/DS surgery.

### Surgical Technique

During the study period, four types of conversion were performed at our institution: SG to either short BPL RYGB, BPD/DS, long BPL RYGB, or RSG. The short BPL RYGB consistent of an antecolic alimentary limb of 150 cm with a linear gastro-jejunostomy and a biliopancreatic limb of 50 cm, whereas the long BPL RYGB had a biliopancreatic limb of 150 cm and an alimentary limb of 100 cm. The jejuno-jejunal mesenteric defect was closed routinely. The BPD/DS was created by a 150 cm alimentary limb with a duodeno-ileostomy and a common channel of 100 cm. The RSG was calibrated over a 32 to 35 French bougie, and the proximal staple line was routinely reinforced with an absorbable running suture.

### Postoperative Outcomes

All patients underwent regular follow-up visits where vital sings, weight change, medical problems, current medication, and laboratory values regarding micronutrients were assessed. Weight outcomes for each group were recorded as mean initial weight and body mass index (BMI), and changes were noted as percentage of total weight loss (%TWL), excess weight loss (%EWL), and as ΔBMI. Associated medical problems evaluated pre- and postoperatively were reported in accordance to the executive summary of American Society for Metabolic and Bariatric Surgery (ASMBS) outcome reporting standards [20].

### Statistical Analysis

The statistical analysis was conducted using GraphPad Prism version 9 and StataMP 17. Continuous and categorical data were described using mean and standard deviation as well as counts and percentages, respectively. Data comparison was performed applying either one-way ANOVA or Kruskal–Wallis test where applicable or Fisher’s exact test. A statistically significant value for *p* was considered to be < 0.05.

## Results

### Patients

Over a time period of 14 years, between 2006 and 2019, 549 SGs were performed, and a total of 84 patients (15.3%) underwent a conversion. Seventy-one patients had a minimal follow-up of 24 months after conversion and were included in the final analysis. The follow-up rates at 5 and 10 years before the conversion were 100% and 90%, respectively. The mean follow-up time after conversion was 5.1 ± 3.1 years. 74.6% (53/71) of the study population were female, and the mean age at conversion was 51.9 ± 10.1 years. The pre-revisional BMI was lowest before short BPL RYGB followed by long BPL RYGB, RSG, and BPD/DS (*p* ≤ 0.001). Before conversion, GERD was present in 89.3% and 82.4% of patients undergoing a short BPL RYGB or a long BPL RYGB and in 21.1% and 57.1% undergoing a BPD/DS and RSG (*p* ≤ 0.001). Table 1 summarizes the demographic factors and the pre-revisional data.

**Table 1:** Patient characteristics and reasons for conversion

	Study cohort	Short BPL RYGB	BPD/DS	Long BPL RYGB	RSG	*p* value
	*n* = 71	*n* = 28	*n* = 19	*n* = 17	*n* = 7	
Sex						
Female – *n* (%)	53 (74.6)	22 (78.6)	17 (89.5)	10 (58.8)	4 (57.1)	0.108
Age (year) – mean (SD)	51.9 (10.1)	53.2 (9.1)	47.5 (9.1)	56.1 (11.2)	48.1 (10.2)	0.043
Initial weight (kg) – mean (SD)	128.9 (26.2)	114.4 (12.7)	140.2 (29.7)	137.5 (29.7)	135.3 (25.6)	0.003
Initial BMI (kg/m^2^) – mean (SD)	46.2 (7.8)	42.4 (4.3)	51.5 (8.8)	47.2 (8.8)	44.7 (5.4)	0.003
Lowest weight (kg) – mean (SD)	93.1 (21.5)	80.7 (15.4)	105.0 (17.2)	97.9 (25.7)	98.3 (20.5)	0.001
Lowest BMI (kg/m^2^) – mean (SD)	33.4 (6.8)	29.7 (4.5)	38.7 (5.1)	33.7 (8.3)	32.7 (6.0)	< 0.001
Pre-revisional weight (kg) – mean (SD)	106.2 (24.2)	90.8 (16.4)	118.5 (17.3)	112.5 (26.3)	119.0 (31.5)	0.002
Pre-revisional BMI (kg/m^2^) – mean (SD)	38.0 (7.5)	33.6 (5.2)	43.7 (5.6)	38.6 (7.8)	39.1 (8.1)	< 0.001
GERD – *n* (%)	47 (66.2)	25 (89.3)	4 (21.1)	14 (82.4)	4 (57.1)	< 0.001
Hypertension – *n* (%)	34 (47.9)	11 (39.3)	12 (63.2)	7 (41.2)	4 (57.1)	0.394
Diabetes – *n* (%)	15 (21.2)	5 (17.8)	6 (31.6)	4 (23.5)	0	0.372
Reasons for conversion						
GERD – *n* (%)	24 (33.8)	17 (60.7)	0	5 (29.4)	2 (28.6)	< 0.001
IWL – *n* (%)	23 (32.4)	3 (10.7)	15 (79.0)	2 (11.8)	3 (42.8)	< 0.001
IWL + GERD – *n* (%)	22 (31.0)	6 (21.4)	4 (21.0)	10 (58.8)	2 (28.6)	0.047
Kinking/stenosis – *n* (%)	2 (2.8)	2 (7.2)	0	0	0	0.594

*Short BPL RYGB*, short biliopancreatic limb Roux-en-Y gastric bypass; *BPD/DS*, biliopancreatic diversion with duodenal switch; *Long BPL RYGB*, long biliopancreatic limb Roux-en-Y gastric bypass; *RSG*, re-sleeve gastrectomy; *SD*, standard deviation; *BMI*, body mass index; *GERD*, gastroesophageal reflux disease; *IWL*, insufficient weight loss

### Conversion

Patients underwent conversion to either short BPL RYGB (*n* = 28, 39.4%), BPD/DS (*n* = 19, 26.8%), long BPL RYGB (*n* = 17, 23.9%), or RSG (*n* = 7, 9.9%). Sixty-eight out of 71 (95.8%) procedures were performed laparoscopically. One patient had a planned open approach, and two patients had to be converted from laparoscopic to open due to adhesions from multiple previous abdominal operations. Concomitant procedures were performed depending on preoperative findings. Twenty-six out of 71 patients (36.6%) had a concomitant cholecystectomy, 35/71 (49.3%) had a hiatal hernia repair, and 5/71 (7.0%) received an abdominal hernia repair. Indications for conversion were GERD (24/71, 33.8%), IWL (23/71, 32.4%), a combination of both (22/71, 31.0%), or kinking/stenosis of the sleeve (2/71, 2.8%) (Table 1).

### Weight Loss During Study Period

**Fig. 1:**
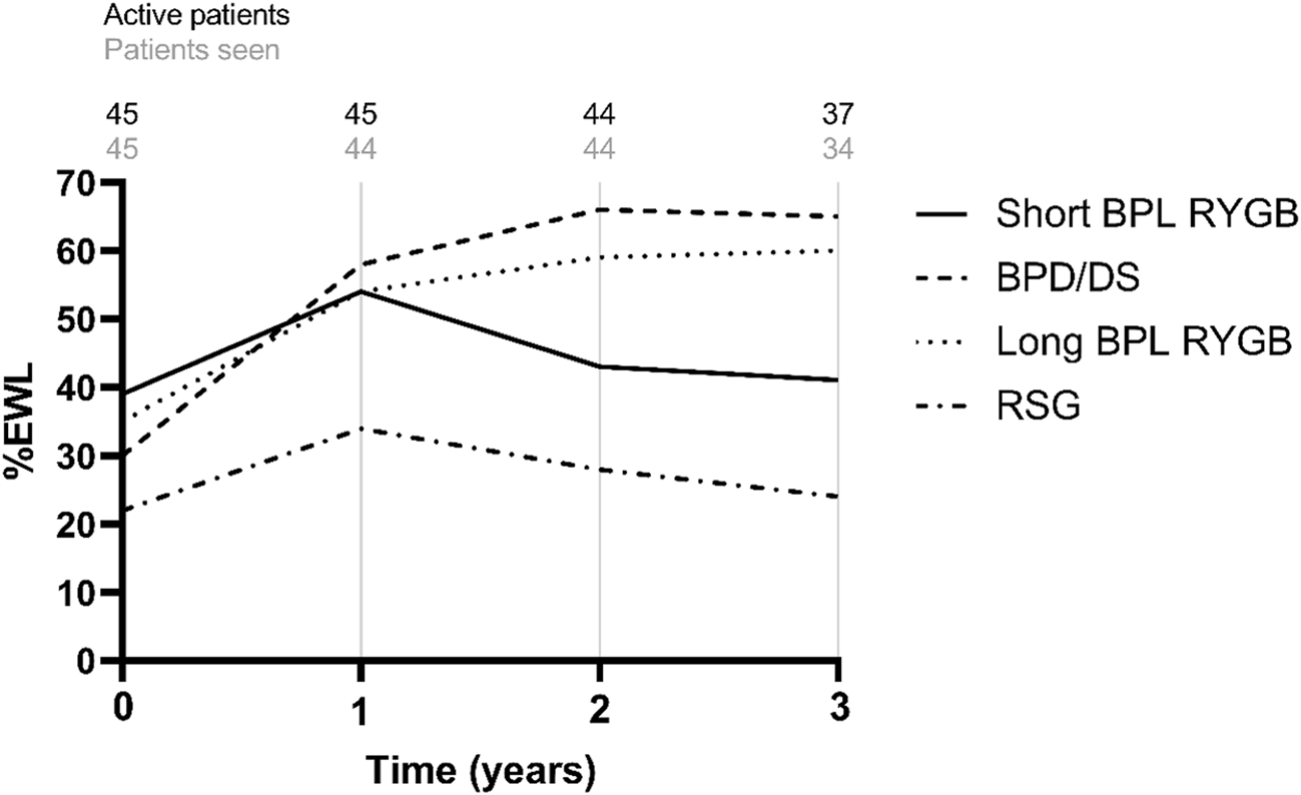
Additional weight loss after conversion at 1, 2, and 3 years for patients with IWL and IWL + GERD as indication for conversion. Short BPL RYGB = short biliopancreatic limb Roux-en-Y gastric bypass; BPD/DS = biliopancreatic diversion with duodenal switch; long BPL RYGB = long biliopancreatic limb Roux-en-Y gastric bypass; RSG = re-sleeve gastrectomy; % EWL = percentage of excess weight loss

**Fig. 2:**
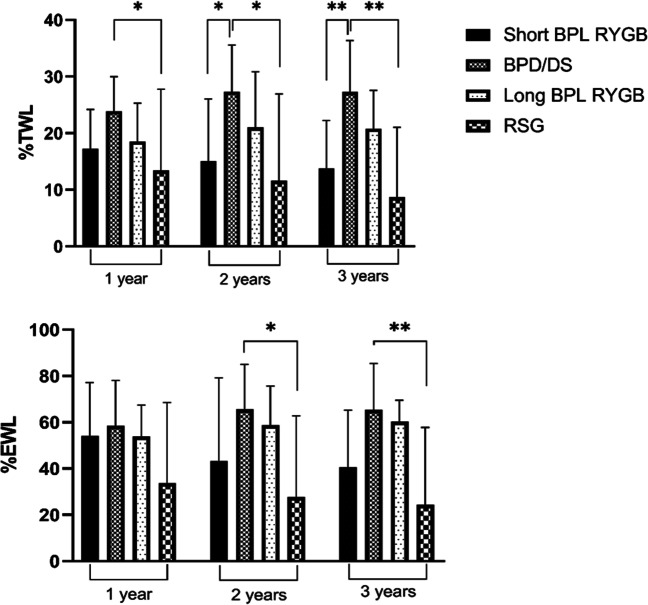
Comparison of additional weight loss after conversion at 1, 2, and 3 years for patients with IWL and IWL + GERD as indication for conversion. Short BPL RYGB = short biliopancreatic limb Roux-en-Y gastric bypass; BPD/DS = biliopancreatic diversion with duodenal switch; long BPL RYGB = long biliopancreatic limb Roux-en-Y gastric bypass; RSG = re-sleeve gastrectomy; %TWL = percentage total weight loss; %EWL = percentage excess weight loss; *p ≤ 0.05; **p ≤ 0.01

Only patients with conversion due to IWL or IWL + GERD were included in the analysis of weight change (Table 2). The overall %TWL and %EWL were highest after BPD/DS and long BPL RYGB reaching statistical significance compared to RSG. Between the other groups, there was no statistically significant difference. Figure 1 depicts the weight loss changes 1, 2, and 3 years after conversion. After that period, the percentage of patients with available follow-up data dropped below 80%. Patients undergoing BPD/DS had a statistically significantly greater additional %EWL compared to RSG. There was no statically significant difference between BPD/DS and long BPL RYGB or short BPL RYGB and neither between short and long BPL RYGB (Fig. 2). Three years after conversion, patients in the BPD/DS group had the highest additional %TWL and %EWL followed by long BPL RYGB, short BPL RYGB, and RSG (Table 2).

**Table 2:** Weight loss during study period for patients with IWL or IWL + GERD as the reason for conversion

	Short BPL RYGB	BPD/DS	Long BPL RYGB	RSG	*p* value
	*n* = 9	*n* = 19	*n* = 12	*n* = 5	
Total					
% TWL – mean (SD)	28.0 (9.6)	36.6 (10.0)	32.9 (7.0)	20.0 (7.3)	0.004
% EWL – mean (SD)	60.4 (17.6)	73.5 (16.1)	72.0 (8.8)	43.5 (15.7)	0.001
Weight loss/BMI changes at 1 year					
Additional % TWL – mean (SD)	17.2 (7.0)	23.9 (6.1)	18.5 (6.8)	13.4 (14.4)	0.027
Additional % EWL – mean (SD)	54.2 (23.0)	58.5 (19.7)	53.9 (13.6)	33.8 (34.7)	0.156
Additional Δ BMI – mean (SD)	6.5 (2.8)	10.5 (2.9)	7.8 (4.3)	6.1 (7.1)	0.039
Weight loss/BMI changes at 2 years					
Additional % TWL – mean (SD)	15.0 (11.0)	27.4 (8.2)	21.0 (9.8)	11.6 (15.3)	0.006
Additional % EWL – mean (SD)	43.3 (35.8)	65.7 (19.3)	58.7 (16.9)	27.8 (35.0)	0.016
Additional Δ BMI – mean (SD)	5.8 (4.3)	12.2 (4.5)	9.0 (6.0)	5.5 (7.8)	0.016
Weight loss/BMI changes at 3 years					
Additional % TWL – mean (SD)	13.8 (8.5)	27.3 (9.0)	20.8 (6.7)	8.7 (12.3)	0.001
Additional % EWL – mean (SD)	40.5 (24.8)	65.5 (20.0)	60.4 (9.1)	24.4 (33.4)	0.005
Additional Δ BMI – mean (SD)	5.3 (3.4)	12.2 (5.0)	8.3 (3.5)	3.5 (5.1)	0.001

*Short BPL RYGB*, short biliopancreatic limb Roux-en-Y gastric bypass; *BPD/DS*, biliopancreatic diversion with duodenal switch; *Long BPL RYGB*, long biliopancreatic limb Roux-en-Y gastric bypass; *RSG*, re-sleeve gastrectomy; *%TWL*, percentage total weight loss; *%EWL*, percentage excess weight loss; *BMI*, body mass index; *SD*, standard deviation

### Postoperative Outcomes

A total of 5/71 patients (7.0%) had major (Clavien Dindo ≥ III) postoperative 30-day complications (Table 3) [21]. All of them occurred after a gastric bypass procedure. The most severe complication was a pancreatitis due to an incomplete obstruction at the entero-enterostomy necessitating intensive care. Two patients had a reoperation due to an early internal hernia, one patient needed a jejunal feeding tube because of a stenosis of the gastro-jejunal anastomosis, and another patient needed surgical wound revision in local anesthesia caused by postoperative bleeding.

**Table 3:** Postoperative outcomes

	Short BPL RYGB	BPD/DS	Long BPL RYGB	RSG	*p* value
	*n* = 28	*n* = 19	*n* = 17	*n* = 7	
30-day complications –* n* (%)					
Clavien Dindo IVa	1 (3.6)	0	0	0	1.000
Clavien Dindo IIIb	1 (3.6)	0	1 (5.9)	0	0.786
Clavien Dindo IIIa	2 (7.1)	0	0	0	0.594
Clavien Dindo II	5 (17.9)	0	1 (5.9)	0	0.163
Clavien Dindo I	0	0	2 (11.8)	0	0.165
Mid-term complications – *n* (%)					
Marginal ulcer	2 (7.1)	0	1 (5.9)	0	0.716
Stricture	1 (3.6)	1 (5.3)	0	0	1.000
Leakage	1 (3.6)	0	0	0	1.000
Dumping	8 (28.6)	1 (5.3)	2 (11.8)	0	0.130
Reoperation	8 (28.6)	3 (15.8)	1 (5.9)	0	0.173
Internal hernia	4 (14.3)	1 (5.3)	1 (5.9)		0.660
Reversal	0	2 (10.5)	0	0	0.233
IWL	3 (10.7)	0	0	0	0.330
CBDS	1 (3.6)	0	0	0	1.000
Persistence of AMP – *n* (%)					
GERD	4 (14.3)	5 (26.3)	4 (23.5)	2 (28.6)	0.665
Hypertension	9 (32.1)	9 (47.4)	3 (17.7)	4 (57.1)	0.170
Diabetes	7 (25.0)	1 (5.3)	1 (5.9)	0	0.169

*Short BPL RYGB*, short biliopancreatic limb Roux-en-Y gastric bypass; *BPD/DS*, biliopancreatic diversion with duodenal switch; *Long BPL RYGB*, long biliopancreatic limb Roux-en-Y gastric bypass; *RSG*, re-sleeve gastrectomy; *IWL*, insufficient weight loss; *CBDS*, common bile duct stones; *AMP*, associated medical problems; *GERD*, gastroesophageal reflux disease

Regarding mid-term complications such as marginal ulcer, stricture, leakage and dumping, there was no significant difference between groups (Table 3). During the study period, 12/71 patients (16.9%) required a further operation after conversion for internal herniation, IWL, and CBDS. Two patients in the BPD/DS group suffered from chronic diarrhea, which did not respond to medical treatment, and malnutrition and required a small intestine interposition to reduce malabsorption. Gastric bypass procedures lead to a resolution of GERD in 79.5%. There was no statistically significant difference regarding the persistence of associated medical problems (i.e., GERD, HTN, and T2D) between procedures.

## Discussion

The main findings of this study include a conversion rate of 15.3% after an average of 5.2 years after SG for mainly GERD (33.8%) and IWL (32.4%) or a combination of both factors (31.0%). Further, we found that the greatest additional weight loss was reached after a conversion with an additional hypo-absorptive component such as BPD/DS or long BPL RYGB. Conversion to a short BPL RYGB led to an initial weight loss which was not sustainable and RSG only led to a minimal additional EWL. After conversion to a RYGB procedure, the resolution of GERD symptoms was 79.5%. Severe short-term complications happened in 7% of patients with no statistical difference between groups.

The above-described conversion rate corresponds to current literature with an overall revision rate of 10.4% increasing to 22.6% if only patients with a follow-up ≥ 10 years are considered [17]. Felsenreich et al. even noted a conversion rate of 49.1% from SG to RYGB with a follow-up of 15 years [22]. However, a significant number of patients show a satisfying outcome after SG as a stand-alone procedure with a long-term percentage excess body mass index loss between 51.0 and 54.0% and a significant improvement of related associated medical problems. Nevertheless, the frequently observed problems of IWL and de novo GERD led to a reoperation rate of 19.2% [23]. It is therefore crucial to inform all patients prior to a SG of the possible complications such as IWL/WR or GERD. It is essential to identify those patients and evaluate conversion before a clinically relevant relapse of associated medical problems occurs. There are various options for conversion of the SG depending on the indication for conversion. In the event of IWL and confirmed sleeve dilatation, a meta-analysis including 196 patients undergoing RSG described a pooled mean EWL of 61.5% after 1 year [24]. However, Cheung et al. found a decline in EWL from 68.0% at 1 year to 44.0% at 2 years after RSG corresponding to our own results of a total EWL of 43.5% [25]. The additional EWL was even lower with 24.4% after 3 years. Therefore, RSG does not seem to be the ideal option for conversion in case of IWL. After a conversion to RYGB, Abdemur et al. found 76.5% total EWL and 30.9% additional EWL with a mixed indication for conversion including GERD [26]. In a subgroup analysis of patients with IWL, D’Urso et al. reported 50.8% total EWL at 1 year decreasing to 45.3% at 3 and 33.8% at 5 years[18]. In our cohort, we similarly saw that a short BPL RYGB led to an initial additional weight loss, which was, however, not sustainable. Therefore, patients with IWL do not seem to profit from such a conversion and seem to need an additional hypo-absorptive component in their bariatric conversion. Revisional procedures with different biliopancretic limb (BPL) lengths have been investigated, and long BPL type procedures were shown to have a significantly higher additional EWL lasting for more than 3 years, while in short BPL type procedures, such as proximal Roux-en-Y gastric bypass, the significance only persisted for 2 years [27]. Andalib et al. and Shimon et al. reported higher TWL after BPD/DS (14.0%; 26.3%) compared to RYGB (10.1%; 18.8%) [8, 28]. Correspondingly, we saw the highest additional weight loss after BPD/DS and long BPL RYGB. However, two patients in the BPD/DS group required a reversal due to chronic diarrhea and malnutrition. In literature, similar results can be found with a small number of patients suffering from severe malnutrition and steatorrhea after BPD/DS and long BPL RYGB [8, 27, 28]. Therefore, a careful patient selection and stringent postoperative follow-up regime is mandatory in case of conversion to BPD/DS to avoid severe side effects.

For GERD as an indication for conversion, RYGB is an effective procedure leading to a remission in 74.0 to 91.3% of patients [29, 30].

In regard to resolution of associated medical problems, BPD/DS shows satisfying results with remission rates of T2D of up to 94.0% and up to 87.5% for HTN [31, 32]. For RYGB, remission rates for T2D of 57.0% and HTN of 44.4% have been found [28, 33]. In our cohort, we have noted a relatively low remission rate of T2D after RYGB of 11.2%, whereas after BPD/DS, it was at 83.3%. Regarding HTN, remission occurred in 33.3% of patients after RYGB and in 25.0% of patients after BPD/DS. However, the incidence of preoperative associated medical problems was relatively low, and the interpretation of the analysis’ results for the four subgroups must, therefore, be done carefully.

Thirty-day complications after conversion to RYGB have been noted between 3.3 and 16.4% [18, 29]. The accumulation of complications after RYGB procedures compared to the other groups in this cohort is most likely a coincidence due to the small sample size, since they are the predominantly performed procedures in our clinic. Andalib et al. compared conversion to RSG, RYGB, and BPD/DS and found a 90-day complication rate of 7.4% [8]. We have seen similar results with 7.0% short-term complications without any statistically significant difference between procedures. Thus, the safety aspect of conversional surgery after SG should not affect the choice of the suitable procedure, since there is no statistical significance amongst them.

Study limitations include the retrospective analysis of prospectively collected data and the small sample size with four different revisional procedures as subgroups. At our institution, we have more than 25 years of experience with BPD/DS and 19 years with SG as a primary stand-alone procedure; however, SG accounts for less than 15.0% of all primary procedures. Yet, the long follow-up time and high follow-up rate of this series support our data.

## Conclusion

Conversion to BPD/DS leads to a significantly higher weight loss than RSG and short BPL RYGB. However, a close follow-up is needed to detect possible nutritional problems. Long BPL RYGB also led to relevant additional weight loss without any severe mid-term nutritional complications and is a valid alternative to BPD/DS. RYGB procedures prompted a good symptom control in patients suffering from GERD.

## Data Availability

The data that support the findings of this study are not openly available due to reasons of sensitivity and are available from the corresponding author upon reasonable request.
